# DWARF – a data warehouse system for analyzing protein families

**DOI:** 10.1186/1471-2105-7-495

**Published:** 2006-11-09

**Authors:** Markus Fischer, Quan K Thai, Melanie Grieb, Jürgen Pleiss

**Affiliations:** 1 Institute of Technical Biochemistry, University of Stuttgart, Allmandring 31, D-70569, Germany; 2 Honig Lab, Dept. of Biochemistry and Molecular Biophysics, Columbia University, 1130 St. Nicholas Ave, NY10032, USA

## Abstract

**Background:**

The emerging field of integrative bioinformatics provides the tools to organize and systematically analyze vast amounts of highly diverse biological data and thus allows to gain a novel understanding of complex biological systems. The data warehouse DWARF applies integrative bioinformatics approaches to the analysis of large protein families.

**Description:**

The data warehouse system DWARF integrates data on sequence, structure, and functional annotation for protein fold families. The underlying relational data model consists of three major sections representing entities related to the protein (biochemical function, source organism, classification to homologous families and superfamilies), the protein sequence (position-specific annotation, mutant information), and the protein structure (secondary structure information, superimposed tertiary structure). Tools for extracting, transforming and loading data from public available resources (ExPDB, GenBank, DSSP) are provided to populate the database. The data can be accessed by an interface for searching and browsing, and by analysis tools that operate on annotation, sequence, or structure. We applied DWARF to the family of α/β-hydrolases to host the Lipase Engineering database. Release 2.3 contains 6138 sequences and 167 experimentally determined protein structures, which are assigned to 37 superfamilies 103 homologous families.

**Conclusion:**

DWARF has been designed for constructing databases of large structurally related protein families and for evaluating their sequence-structure-function relationships by a systematic analysis of sequence, structure and functional annotation. It has been applied to predict biochemical properties from sequence, and serves as a valuable tool for protein engineering.

## Background

In the last decades large amounts on biological data were accumulated and the high-throughput methods in the fields of genomics and proteomics create an ever-increasing rate of high dimensional data on sequence, structure, and function of biological systems. The systematic analysis of these data provides the opportunity to gain a novel level of the understanding of complex biological systems. However the data are highly diverse and widely scattered across hundreds of databases and thus are difficult to exploit. Therefore methods have to be developed that allow the integration of these diverse data in a consistent way. The emerging field of integrative bioinformatics has been implemented to cope with this problem. By providing the essential methods to integrate, manage, and analyze these data, the integrative bioinformatics allows to gain new insights and a deeper understanding of complex biological systems.

To fully explore the sequence-structure-function relationships within a protein fold family, integrative bioinformatics has proven in recent studies to be a powerful tool. Already 10 years ago Cousin et al. started to organize publicly available data on genes, mutants, biochemical and pharmacological data for the protein family of acetylcholinesterases in a database system [1]. Using this data in combination with structure prediction it was possible to infer a model for the association of catalytic and structural subunits [2]. In another integrative bioinformatics study Barth et al. revealed for epoxide hydrolases that three loop regions can be correlated with the substrate specificity of this enzyme family [3].

Because integration of widely distributed data is prerequisite to their analysis, several approaches for data integration have been investigated: linked, indexed data connect flat file databases using the World Wide Web (WWW) like SRS [4] or Entrez [5,6], or federated database systems which integrate heterogeneous database systems by a central query interface (examples of the latter are OPM*QS [7] and the Genome Database, GDB [8]). In contrast, data warehouse systems (like the Integrated Genomics Database, IGD [9] and MetaFam [10]) provide a tight data integration by a common data schema and periodically load all data into a central repository. Thus, the concept of data warehousing helps to overcome two major limitations of distributed database systems: inconsistency of data and time consuming or incomplete queries caused by server restrictions.

To facilitate the analysis of sequence-structure-function relationships within a protein fold family, information on protein sequence, structure, and functional annotation are provided by various public databases. However, functional annotation is often incomplete and sometimes inconsistent because it is manually integrated from publications into the database by the curator of the database or the authors of the entry. By integrating these public data into a single database the functional annotation can be validated and enriched by annotation transfer within well-defined sequence families. Pre-classified data on sequence clusters [11] or structural domains and architectures [12,13] provide a valuable starting point for assembling sequences and structures. The build-up process of a protein family database includes four steps. (1) Data on sequence and structure are integrated into the underlying data schema. (2) Proteins are assigned to superfamilies and homologous families based on sequence similarity. (3) By comparing multisequence alignments and phylogenetic trees the classification is validated and annotations are enriched. (4) The validated data provide a reliable basis for analyzing the protein family and to derive hypotheses for the sequence-structure-function relationship within the protein family.

One of the largest family of structurally related proteins are the α/β-hydrolases, which catalyze a broad variety of chemical reactions and accept highly different substrates [14]. Despite their diversity in sequence they all share a highly conserved catalytic triad: a nucleophile (Ser, Cys, Asp), a histidine, and an acid (Asp, Glu). Ten years ago [15], structure data for only five homologous proteins were known. In the meantime the protein structure classification database SCOP [13] (release 1.69) lists 534 different structure data sets classified in 32 sequence families. The family includes carboxylic acid ester hydrolase, lipid hydrolase, thioester hydrolase, peptide hydrolase, haloperoxidase, dehalogenase, epoxide hydrolase and C-C bond breaking enzymes, and even members that have lost the catalytic triad like the cell adhesion proteins gliotactin, glutactin or neuroligin [1,16]. Thus, the α/β-hydrolase fold is a typical example for a divergent evolution where the development of different functions evolved from a common ancestor.

We describe here the data warehouse system DWARF, which is dedicated to the analysis of protein families. So far there is no integrative approach described, that focuses to the analysis of the sequence-structure-function relationships within a single protein fold family. In an iterative process sequence clusters are generated and functional annotation is enriched by an automated annotation transfer. The validated data content of DWARF facilitates the analysis of sequence-structure-function analysis and guides protein engineering within protein families. We applied the DWARF data warehouse to the family of α/β-hydrolases, to hostthe Lipase Engineering Database, which has proven to be a powerful tool in analyzing the protein fold family of α/β-hydrolases.

## Construction and content

### Source data

DWARF was designed to store data on protein sequences, structures, functional annotations, and a hierarchy of families. Protein sequence related data are extracted from GenBank [17], though the data model is designed to hold also sequence data from other data sources. For each GenBank entry the sequence, position-specific features, the source organism, the source database, available accession codes, and the protein name are extracted.

The main data source for protein structure coordinates is ExPDB [18], since DWARF is designed to store single structure chains. ExPDB is derived from the Protein Data Bank (PDB) and provides an individual file for each single protein chain. Beside the coordinates, additional data on experimental resolution, authors, and publication year are extracted. The monomer sequences were derived from the ATOM section of the coordinate files.

### Relational data model

**Figure 1:**
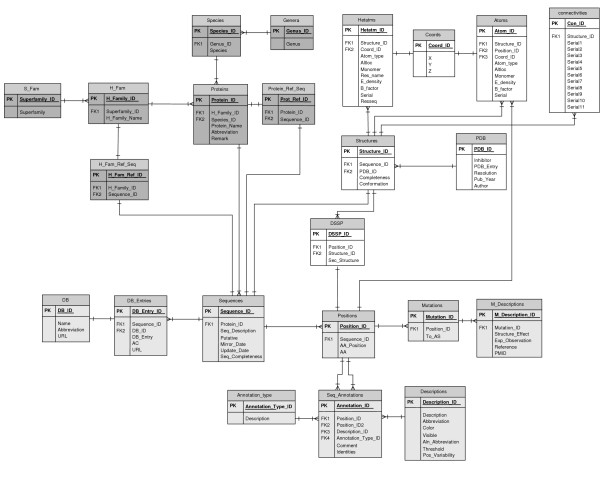
Conceptual data schema for DWARF, using Logical Data Structure (LDS) notation. Each box represents an entity, which is implemented as a database table. Primary Key attributes that identify each data instance of the entity are underlined. Entities associated with the protein are shaded in dark grey on the upper left, protein structure in white on the upper right, and entities describing protein sequence properties are shaded in light grey. Lines between the entities describe existing relationships.

The data model is implemented in Firebird [19], an open source relation database management system (RDBMS). It is divided in three major sections (Fig. 1): entities describing (1) the protein, (2) the protein sequence, and (3) the protein structure. The data schema was developed for integrating data on protein families sharing a common fold.

#### Protein section

Proteins are stored in **Proteins **and are defined by the source organism (**Species **and **Genus**). A protein entry can hold several sequence entries, because often slightly different sequences are available from different authors or the sequences depend on the source strain. Thus, a single protein entry is assumed to hold multiple sequences if their sequence identity is higher than 98%. In the case of multi-domain proteins only the domain sharing the same fold is used for determination of sequence identity. For each protein entry a reference sequence entry is defined in **Protein_Ref_Seq**. Based on sequence identity, the hierarchy of protein entries is defined by three levels: homologous superfamilies (**S_Fam**), homologous families (**H_Fam**), and proteins (**Proteins**). For each homologous family a reference sequence is defined in **H_Fam_Ref_Seq**.

#### Sequence section

Sequence entries (**Sequences**) are checked for duplicates and a set of non-redundant protein sequences is created. The source database entries are stored in **DB_Entries **for each sequence entry. Each amino acid is stored as a single data set (**Positions**), thus annotations (**Seq_Annotations**) can be easily mapped on single amino acids. Three types of annotations are defined in **Annotation_type**: single position specific, pairs of positions, and regions. The feature properties are defined in **Descriptions **and can be adjusted to the needs of the investigated protein family. Annotation information for a single sequence is merged from different source databases.

#### Structure section

The protein structure section is adapted from the PDB data model [20] to describe experimentally determined protein structures as well as theoretical models. Each monomer is stored as a single structure entry (**Structures**). The table **PDB **holds the information on the source of the structure entries. Data on atoms, heteroatoms, coordinates and connectivities is stored in **Atoms**,**Hetatms**, **Coords and Connectivities**, respectively. The sequence corresponding to each structure data set is stored as separate sequence entry. Thus structure-derived properties like secondary structure information (**DSSP**) are mapped on the sequence and consistently annotated structure data sets are generated. This approach also overcomes the problem of sequence length inconsistencies caused by unresolved residues.

### Architecture

The data warehouse was developed on Linux (Fedora Core 3) and is built as a server-client architecture. Scripts for the back-end and query interface were developed in Perl. For the web-based query interface the Apache web server [21] is used. Since the query interface was build as a set of CGI (common gateway interface) scripts, each Internet browser can be used as client software. The usage of the standard Perl CGI modules [22] ensures a high compatibility between scripts and Internet browsers. Some tasks (highlighting or selection of features) have been developed in Javascript. Besides the web-based query interface, the access on SQL level allows to query the underlying data directly. Thus, assisted by the position-specific sequence description, complex queries can be easily performed.

### Data loaders

For the population of the DWARF data tables, data loaders were developed to parse, transform, and load information form the original source data sets. The data loaders are separated into two main categories: (1) sequence specific data loaders for information derived from GenBank [17] and (2) a data loader for protein structure information on coordinates derived from ExPDB [18] and secondary structure information calculated by DSSP [23].

The sequence specific data loaders extract selected information from the GenBank data sets. These data sets have been represented in the NCBI ASN.1 format, but since recently also XML-formatted GenBank data sets are available. Data loaders for either the ASN.1 or the XML Genpept format are provided in DWARF.

The protein structure-specific data loader is designed to parse the PDB formatted ExPDB coordinates data set and the DSSP secondary structure description, which is generated for each protein structure chain.

### Data population

Upon setting up a new protein family database, the initial step of data population consists in selecting seed sequences from classification databases like SCOP [13] or CATH [12], and sequences derived by keyword searches in primary sequence databases. These seed sequences are then used as a starting point for iterative BLAST [24] searches to achieve a complete coverage of the known sequence space for the protein family. The BLAST searches in the GenBank [17] non-redundant protein database are performed by an automated data extraction system. From the start, seed sequences are assumed to represent a homologous family each. Then new hits are automatically assigned to one of the existing homologous families by a standalone BLAST search against all sequences already present in the data warehouse. For hits that are not sufficiently similar to an already existing entry, a new superfamily or homologous family is created. During the data population process the classification of sequences into homologous families and superfamilies is permanently controlled, new families are created, or homologous families are merged.

The resulting GenBank hit entries are retrieved for further processing. Information on organism, original database entries, sequence features, mutations, and sequence are extracted and transformed into the DWARF data model using the sequence data loaders. For sequences that are linked to structure entries, header information and atom coordinates are extracted from ExPDB [18]. For each structure entry the secondary structure information is calculated using DSSP [23].

The protein family databases are updated regularly by an automated Perl script. To update a database, a BLAST search against the current version of the non-redundant sequence database GenBank for each sequence entry is performed. For a sequence entry referring to a new entry in the PDB, structure information is updated as well. New sequences and structure entries are classified into homologous families and superfamilies based on their sequence similarity.

### Web interface

Since DWARF is designed as an in-house repository assisting local analysis rather than to be a public accessible resource, there are two interfaces: a browser available to the public and a set of data analysis tools available to the local curator. For public access a HTML-based WWW interface is provided [16], which lists all superfamilies and families stored in the data warehouse. For each family a list of protein entries is given. Protein entries are described by source organism, protein name, and description, and are linked to their source entry at GenBank [17]. For structure data sets annotated protein structure monomers are available in PDB format [20]. For each family, consistently annotated multisequence alignments and phylogenetic trees are generated, and raw sequence data sets are provided. BLAST searches can be performed to identify sequence similarities and a SQL interface allows access to the underlying relational database.

For the database curator, additional tools for validation and enrichment of the database are available. Protein name and sequence identifiers can be edited and sequences can be added or removed from protein families, as derived from multisequence alignments using CLUSTALW 1.83 [25]. Alignments can be restricted to single domains in case of multidomain proteins. For each multisequence alignment a similarity score is calculated using PLOTCON of the EMBOSS package [26]. Features for sequences represented by the multisequence alignment are annotated and the sequence identifiers are linked to the editing script. Based on the multisequence alignment a neighbor-joining tree is displayed. These trees are represented by the CGI based tree visualization tool PHYLODENDRON [27]. The neighbor-joining based phylogenetic trees are calculated interactively. A reclassification tool for individual sequences is linked to sequence identifiers within the multisequence alignments and the phylogenetic trees. To validate and enrich annotations extracted from the primary sequence databases, a workbench system allows to add, to delete, or to transfer annotation between sequences.

### Annotation workflow

The annotation workflow is separated into two steps: (1) validation of the protein classification followed by (2) the validation and enrichment of sequence annotations.

The classification of protein sequences is verified by multisequence alignments for homologous and superfamilies, as well as by evaluating phylogenetic trees. Highly conserved sequence patterns serve as family descriptors and guide the reclassification of individual sequences, addition of new families or merging existing families.

Based on the consistent classification, reliable multisequence alignments for the families were used for validation, correction, and enrichment of the annotation retrieved from SWISSPROT [28] and PIR [29], information from literature, or information derived from in-house experiments. Annotated multisequence alignments serve as a quality control of annotation information and of the alignment: if both are correct and consistent, structurally and functionally relevant residues should align.

Validated and enriched annotation is mapped on representative sequences of each homologous family using the annotation workbench. Subsequently an automated sequence annotation system transfers the annotation on all members within the corresponding homologous family based on multisequence alignments. Rules for the annotation transfer can be defined for each annotation based on amino acid composition and percentage of coverage for expected amino acids within an alignment column.

## Utility

DWARF is a relational integrated data warehouse of information on protein structure, sequence, and functional annotation, and is designed to systematically analyze protein families. These analyses can be performed on three different data access levels, providing a maximum flexibility of data retrieval and integration: (1) The HTML-based web resource allows the user to browse the available data on a protein fold family. It provides a sequence based classification, an overview on available sequence and structure data, and a consistent annotation of functionally relevant amino acids that can assist the identification of functionally relevant amino acids. (2) The interactive web interface with access to the underlying database allows to manipulate the data directly without the need of programming. Individual annotations can be incorporated into the analysis by mapping it on sequences within interactively generated multisequence alignments. The modular design of the interactive web interface also allows an easy implementation of individual Perl wrappers for further analysis tools like a HMM profile-profile analysis for α/β-hydrolases. (3) The data access on the SQL level allows complex queries. Assisted by the position-specific data modeling of protein sequences, statistics like the amino acid composition within families or organisms, occurrence of functionally annotated amino acids, or sequence length distributions can be evaluated easily.

## Application

### The Lipase Engineering Database

We applied the DWARF data warehouse system to set up the Lipase Engineering Database. The current release 2.3 now comprises the complete α/β-hydrolase fold family. The Lipase Engineering Database can be browsed by organisms, by protein structures, or by a systematic family classification represented by a phylogenetic tree that is based on distances derived from HMM profile-profile comparisons. Links are available to the NCBI taxonomy server [30] for organisms and corresponding SYSTERS clusters [11] for homologous families, as well as references to the classification of bacterial lipases by Arpigny and Jaeger [31]. Collections of sequences in FASTA format and structures in PDB format can be downloaded for each superfamily and homologous families.

### Classification

To estimate the relationships between the superfamilies of α/β-hydrolases, Hidden Markov Models (HMM) were generated for each homologous family. Distances were inferred by HMM profile-profile comparison based on p-values [32]. These distances were used to create a phylogenetic tree based on the UPGMA method as implemented in the PHYLIP package [33]. According to this tree a systematic nomenclature for α/β-hydrolases was introduced: **abHn.m**, where **abH **is followed by the superfamily number **n**, which is separated from the homologous family number **m **by a dot.

Based on structure and sequence analysis of the oxyanion hole, α/β-hydrolases were classified into three classes, the GGGX-, GX- and an additional Y-class.

The GGGX-class consists of 4 superfamilies with known protein structures, where the oxyanion hole-forming residue is located in a well conserved GGG pattern, which is followed by a conserved hydrophobic amino acid X. The backbone amide of glycine G preceding the residue X forms the oxyanion hole. Based on HMM profile-profile comparisons, two further superfamilies (abH2, abH5) that contain no protein structure data were also assigned to the GGGX class, because they show significant similarity in the oxyanion hole region (see Table 1). The GGGX- class comprises the families abH1-abH6.

**Table 1:** Oxyanion hole region for representative sequences of the GGGX class.

**Family**	**Accession code**	**Oxyanion hole region**
abH1	1MX1F	KNRLPVMVWIHGG**G**LMVGAAS
abH2	Q99156	ATNLPVFVWIHGG**G**NLAGNGY
abH2	50546206	-ARLPTVVWIHGG**S**NIEGSIY
abH3	1LPS	-ANLPVMLWIFGG**G**FEVGGTS
abH4	1JJIA	-S--PVLVYYHGG**G**FVICSIE
abH5	AAC50666	-RSRSLIVHFHGG**G**FVAQTSR
abH5	CAF98042	-SSPCIVIHFHGG**G**FVAQTSK
abH6	1JKMA	--VLPGLVYTHGG**G**MTILTTD

The sequence block was extracted from an alignment of representative GGGX sequences. This alignment illustrates the high similarity within the oxyanion hole region for the families abH2 and abH5 (without protein structure entries), and the remaining GGGX class families which include proteins with known structure. The oxyanion hole forming residue is indicated in bold letters.

The GX-class consists of 21 superfamilies with known protein structures, where the oxyanion hole-forming residue X is structurally well conserved and is preceded by a strictly conserved glycine. Based on HMM profile-profile comparisons another six superfamilies (abH7, abH10, abH16, abH17, abH21, abH24) that contain no protein structure data were also assigned to the GX class. They also show significant similarity in the oxyanion hole region. The GX-class comprises the families abH7-abH26, abH31-abH37.

The Y-class consist of 4 superfamilies with known protein structures, where the oxyanion hole is not formed by a backbone amide, but by the hydroxyl group of a tyrosine side chain, which is strictly conserved within the superfamilies. The Y-class comprises the families abH27-abH30.

### Database content

The release 2.3 (July 2006) of the Lipase Engineering Database contains 6138 sequence entries for 4322 proteins and thus covers the whole α/β-hydrolase fold. 36% of these proteins are marked as putative. For 41 protein entries, 167 experimentally determined protein structures comprising 574 structure data sets are available. The sequences were collected by performing single iteration BLAST searches (BLOSUM62 scoring matrix, 10^-10 ^as E-value cutoff) for seed sequences against the non-redundant sequence database GenBank.

The 4322 proteins were assigned to 37 superfamilies and 103 homologous families. The superfamily abH1 is the largest family and comprises 829 protein entries representing 19% of all Lipase Engineering Database protein entries. The three largest superfamilies abH1, abH8 and abH34 comprise 1694 protein entries representing 39% of the Lipase Engineering Database. The length of 93% of all α/β-hydrolase domains is between 181 and 615 amino acids. The minimal and maximal size of the α/β-hydrolase domain is defined by the lipase A from *Bacillus subtilis *(the smallest lipase known, 181 amino acids) and the cocaine esterase from *Xanthomonas citri *(615 amino acids), respectively. Protein entries falling below this length are considered as fragments, since the structure of the lipase A from *Bacillus subtilis *suggests that shorter sequences likely will lack secondary structure elements that are essential for the α/β-hydrolase fold. Protein entries exceeding this length mainly comprise mammalian carboxylesterases that include an additional transmembrane region, as well as putative proteins from *C. elegans *and putative alpha esterases.

**Figure 2:**
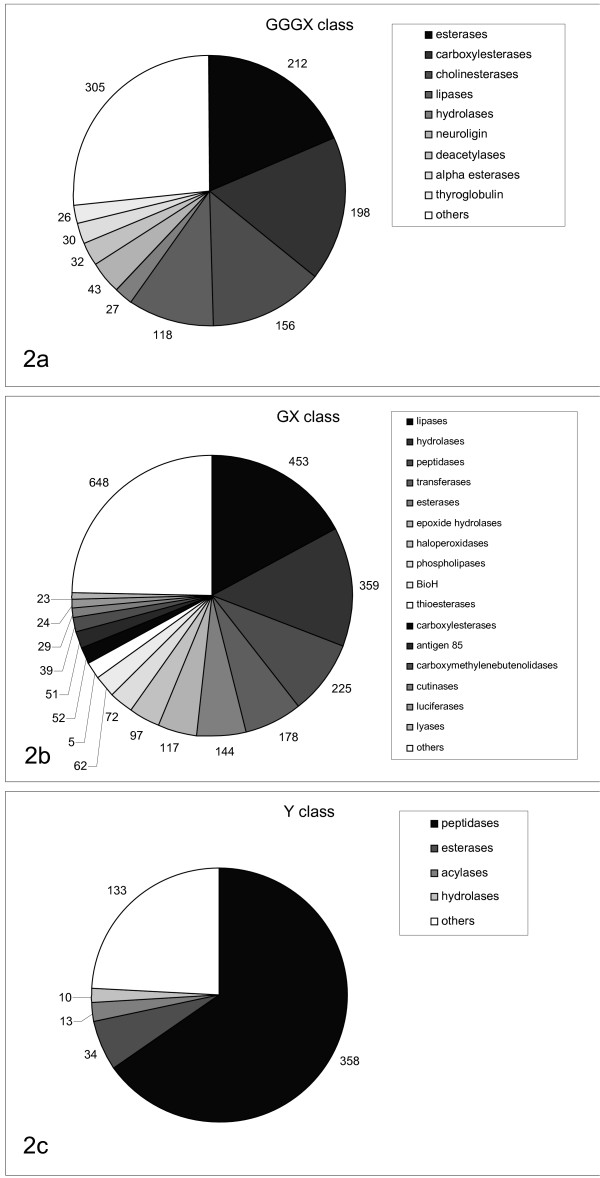
Distribution of protein functions for the three α/β-hydrolase classes GGGX (a), GX (b), and Y (c). Putative proteins with no function assigned and functions with a low percentage were grouped into "others".

The GGGX-class consists of 6 superfamilies and 20 homologous families, containing 1146 protein entries, 1780 sequence entries and 167 structure data sets. This class mainly consists of bacterial esterases, α-esterases, eukaryotic carboxylesterases, bile-salt activated lipases, juvenile hormone esterases, hormone-sensitive lipases, acetylcholinesterases, brefeldin A esterases, and thioesterases (Fig. 2a)

The GX class consists of 27 superfamilies and 79 homologous families, containing 2628 protein entries, 3623 sequence entries and 349 structure data sets. This class mainly consists of bacterial and fungal lipases, eukaryotic lipases (hepatic, lipoprotein, pancreatic, gastric and lysosomal acid lipases), cutinases, phospholipases, non-heme peroxidases, acyltransferases, epoxide hydrolases, haloalkane dehalogenases, deacetylases, hydroxynitril lyases and thioesterases (Fig. 2b).

The Y class consists of 4 superfamilies and 4 homologous families, containing 548 protein entries, 735 sequence entries and 58 structure data sets. This class mainly consists of dipeptidylpeptidasen, propylendopeptidasen and cocaine esterases (Fig 2c).

### Alignments

Fully and consistently annotated multisequence alignments are provided for each superfamily and homologous family of the α/β-hydrolases. In the case of multiple domain proteins, only the α/β-hydrolase domain is represented. For superfamily alignments, all protein entries were selected, which share less than 90% sequence identity. Corresponding homologous families for these representative sequences are indicated by the systematic family names within the superfamily alignments.

For representative Lipase Engineering Database protein entries DNA information was extracted from GenBank [17] covering 94 of 103 homologous families. DNA alignments were created using ClustalW 1.83 [25] and colored by degree of conservation. Only coding regions were used. GenBank accession codes were used as identifiers within the alignments and linked to additional information from the GenBank entry like source organism, locus, topology, translation table, a graphical representation of the coding region, and the original GenBank entry.

## Discussion

The data warehouse system DWARF has been developed to provide a platform for the analysis of the sequence-structure-function relationships within protein families. To facilitate these analyses the integration of publicly available data, in-house data, and information extracted from literature has proven to be a powerful approach of integrative bioinformatics. The integration of data in a local repository assures a fast and complete response to complex queries. The response time is not limited by external server restrictions. The local deposition of the data also allowed us to design a data model that represents a tight integration of semantic similar data like position-specific annotations, which facilitated the automated annotation transfer. Thus we achieved a consistent annotation of family members and a position-specific data modeling of protein sequences, which allows the systematic analysis of protein families on database level.

To provide a high flexibility in accessing the underlying data, three levels of data access were implemented: (1) the database content is visualized by static HTML pages and allows the user to browse the database, (2) the interactive web interface serves as tool for analyzing and maintaining the database without the need of programming ability, and (3) the direct access of the data by using complex SQL queries.

### The data warehouse approach

A data warehouse system has several advantages as compared to alternative solutions: (1) Web resources based on indexed flat file databases like SRS [4] or Entrez [6] integrate distributed data sources by extracting specific fields to create cross-references by hyperlinks. These systems represent a loose integration of data but can be easily implemented. However, hyperlinks are not necessarily permanent, and therefore this type of data integration requires to be maintained regularly. Additionally the provided data must be processed before it can be analyzed. (2) Federated database systems like OPM*QS [7] and the Genome Database [8] integrate data by accessing heterogeneous, distributed databases by a central query interface. These systems do not store the data locally but query the original data sources by a middleware. These systems share the advantage to query data sources independent on the sources' data model. However, complex queries can be very time consuming or are responded incompletely because of server restrictions or temporarily unavailable services.

### Protein family databases

Commonly the data warehouse approach is applied to integrate data on whole genomes like the Integrated Genome Database, GDB [9] or to gather information on the complete protein sequence space like MetaFam [10] or Atlas [34] to facilitate classification of protein families or infer protein-protein interactions. DWARF is dedicated to remedy the lack of tools that assist the analysis of single protein families by providing a detailed view on the sequence-structure-function relationships within these families.

The most similar system compared to DWARF is the database system ESTHER [1]. Like the Lipase Engineering Database, ESTHER is specialized on storage, classification, and annotation of sequence data on α/β-hydrolases. ESTHER has been pioneering the integration of protein family data into a single database system. In contrast to DWARF, which uses a relational data model and allows standard SQL statements for data mining, ESTHER is based on the AceDB software. AceDB has been developed to store and analyze data in the framework of the C. elegans genome project and stores data as objects organized in classes. Data can be retrieved by the SQL-like query language AQL. Like by most other database systems, in ESTHER protein sequences are represented as single object, which can make sequence analysis tedious. To facilitate comprehensive position-specific analyses of sequence data DWARF models sequence on an amino acid level.

### Data modeling

DWARF was designed to assist building protein fold family databases providing a consistent annotation of position-specific functionally relevant features. Information extracted from public sources is validated by the biochemical knowledge of the database curator and enriched by a subsequent automated annotation transfer. Therefore the design of the underlying data model was aimed for facilitating intuitive database queries for functional relationships on the amino acid level combining sequence and structure information. Thus we have decided to merge semantic similar types of data like amino acid-specific annotations within single tables, even if they are derived from different sources. Compared to the multidimensional data integration approach [35], which stores similar data from different source separately, we achieve a tighter integration of the data which allows an easier way to query functional relationships.

This tight integration can mean also that fewer changes are needed over the lifetime of the data warehouse compared to a multidimensional approach, where for each new data source the data model has to be adjusted. In DWARF, new data sources can easily be loaded into the DWARF data model by an additional data loader that is specific to the data source.

Though it is common to store the sequence of amino acids as a complete string like in the case of ESTHER [1] or Atlas [34] we store each amino acid as a single entry. We think that the drawback of a lower performance for extracting protein sequences is compensated by the fact that position specific queries can be executed on database level rather to be implemented within applications. Moreover this data modeling assists the annotation and analysis of position specific features.

We also integrated protein structure coordinates within the data model, which allows an easy incorporation of functional annotations within protein structures, analysis of geometry properties, and definition of structural restrains within queries that can be used for the validation of annotation transfer or to identify functional modules.

### Systematic Analysis

DWARF is a versatile integrative bioinformatics tool to build protein family databases and to assist the study of sequence-structure-functions relationships. Beside the family of α/β-hydrolases, DWARF has also been applied to build databases for the protein families of GH16 glycoside hydrolases [36] and cytochrome P450 monooxygenases (in Preparation). These databases provide the basis for a systematic analysis of these protein families. For epoxide hydrolases, a subfamily of the α/β-hydrolases, the comparison of structural differences between epoxide hydrolases and the systematic analysis of loop regions resulted in a new classification scheme. The loop regions were correlated with the substrate specificity of epoxide hydrolases and the classification facilitated the modeling of 80% of all epoxide hydrolases [3].

The database of GH16 glycoside hydrolases has been used for homology modeling of seven glycoside hydrolases from the GH16 family with various substrate specificities. The models predicted the position of functionally relevant amino acids in the substrate-binding site that are similar to the family GH11. This structural comparison indicates evolutionary connections between these two families [36].

## Conclusion

DWARF was designed to facilitate the easy construction of protein family databases and to assist the analysis of sequence-structure-function relationships. The automated annotation transfer guided by biochemical knowledge results in a consistent annotation of the data integrated by the data warehouse system. We have applied the system to systematically compare various protein families to infer substrate specificities and to guide the engineering of optimized enzymes.

## Availability and Requirements

DWARF was developed on Linux (Fedora Core 3). Scripts for the back- and front-end were developed in Perl. The relational DWARF database can be accessed by the SQL interface located at . For the Lipase Engineering Database the web interface created by DWARF is accessible at . As in the previous release, it can be browsed by superfamilies and homologous families. BLAST searches can be performed against all sequence entries. Links are available to consistently annotated multisequence alignments, phylogenetic trees and structure data sets.

## Authors' contributions

MF designed and implemented the data warehouse system. TKQ developed the annotation workbench. MG was responsible for the implementation of the automated annotation transfer. JP was the principal investigator, conceived the project and guided its development. All authors read and approved the final manuscript.
